# Assessment of quality of care among in-patients with postpartum haemorrhage and severe pre-eclampsia at st. Francis hospital nsambya: a criteria-based audit

**DOI:** 10.1186/s12884-016-1219-y

**Published:** 2017-01-13

**Authors:** Alfred Lumala, Peter Sekweyama, Andrew Abaasa, Humphrey Lwanga, Romano Byaruhanga

**Affiliations:** 1 Nkozi Hospital, Kampala, Uganda; 2 Uganda Martyrs University Mother Kevin Postgraduate Medical School, Kampala, Uganda; 3 Medical Research Council, Kampala, Uganda; 4 Mukwaya General Hospital, Kampala, Uganda

**Keywords:** Criteria-based audit, Postpartum haemorrhage, Pre-eclampsia, Uganda

## Abstract

**Background:**

The maternal mortality ratio of Uganda is still high and the leading causes of maternal mortality are postpartum haemorrhage (PPH), severe pre-eclampsia and eclampsia. Criteria-based audit (CBA) is a way of improving quality of care that has not been commonly used in low income countries. This study aimed at finding out the quality of care provided to patients with these conditions and to find out if the implementation of recommendations from the audit cycle resulted in improvement in quality of care.

**Methods:**

This study was a CBA following a time series study design. It was done in St. Francis Hospital Nsambya and it involved assessment of adherence to standards of care for PPH, severe pre-eclampsia and eclampsia. An initial audit was done for 3 consecutive months, then findings were presented to health workers and recommendations made; we implemented the recommendations in a subsequent month and this comprised three interventions namely continuing medical education (CME), drills and displaying guidelines; a re-audit was done in the proceeding 3 consecutive months and analysis compared adherence rates of the initial audit with those of the re-audit.

**Results:**

Pearson Chi-Square test revealed that the adherence rates of 7 out of 10 standards of care for severe pre-eclampsia/eclampsia were statistically significantly higher in the re-audit than in the initial audit; also, the adherence rates of 3 out of 4 standards of care for PPH were statistically significantly higher in the re-audit than in the initial audit.

**Conclusion:**

The giving of feedback on quality of care and the implementation of recommendations made during the CBA including CME, drills and displaying guidelines was associated with improvements in the quality of care for patients with PPH, severe pre-eclampsia and eclampsia.

## Background

PPH is the leading cause of maternal death contributing 19.7% to maternal mortality globally and it affects approximately 2% of all women who give birth [1, 2]. Hypertensive disorders in pregnancy are the second leading cause of maternal mortality contributing 14% to maternal mortality worldwide; Pre-eclampsia stands out among these disorders as a leading cause of maternal and perinatal morbidity and mortality and it is found in 2%–8% of all pregnancies worldwide [3]. Criteria based audit (CBA) is assessing the process component of quality of care provided to patients with a specified condition by comparing the care against explicitly agreed standards [4]. CBA is a method of measuring quality of obstetric care that reliably checks for adherence to guidelines and leads to improvement in quality of care [5].

The CBAs have already been influential in reducing maternal and perinatal mortality in the low income countries, but have not yet been widely used in the low income countries like Uganda [6]. These CBAs have shown positive improvement in the quality of care in some low income counties [7]. This study had an aim of assessing the quality of care given to inpatients with PPH and severe pre-eclampsia in St. Francis Hospital Nsambya as a way of reducing maternal mortality. The specific objective was to determine the improvement in adherence to guidelines during care given to inpatients with PPH and severe pre-eclampsia in St. Francis Hospital Nsambya after implementing recommendations made from an initial audit.

## Methods

St. Francis Hospital Nsambya is a Catholic founded Private-Not-For-Profit tertiary care hospital located in the Southern part of Kampala city, about 3 km from the city centre. It is a tertiary referral hospital with a bed capacity of 361 and an average of 20 deliveries per day.

This was a time series study. After an initial audit of care, findings were presented to staff of the department of obstetrics and gynaecology, recommendations for improvement were made and implemented, then another audit of care was done one month later. The 2 audits were carried out by assessing the adherence to standards of care for severe preeclampsia, eclampsia and PPH. The standards of care were based on WHO guidelines and they are out lined in Table 1 below.

**Table 1:** Standards of care for severe pre-eclampsia, eclampsia and PPH

Standards of care for severe pre-eclampsia/eclampsia	Standards of care for PPH
1. Patients should be seen by a doctor within 1 h (h) of arrival.	1. AMTSL should have been done before PPH is diagnosed
2. Anti-hypertensive therapy should be started within 20 min (min) of diagnosis.	2. IV oxytocin should be given as soon as possible when PPH is diagnosed
3. Urine protein dipstick test should be done within 30 min	3. IV isotonic crystalloid fluids should be given as initial fluid resuscitation when PPH is diagnosed
4. BP should be monitored every 30–60 min when the diastolic BP is ≥110 mm Hg.	4. In case bleeding does not respond to oxytocin, IV ergometrine or misoprostol should be given when PPH is diagnosed
5. The fetal heart rate (FHR) should be monitored every 30 min when the diastolic BP is ≥110 mm Hg.	
6. Magnesium sulfate should be administered within 20 min. of diagnosis	
7. A FBC RFT and LFT should be done within 24 h	
8. Steroid therapy should be given in all pregnancies where the pregnancy is estimated to be 28–34 weeks gestation.	
9. Deep tendon reflexes test and respiratory rate monitoring should be done for 24 h	
10. Caesarean section (CS) should be done in 1h from when decision is made	

In this study, the intervention was presentation of findings of the initial audit and the implementation of the three recommendations made namely; a half hour didactic continuing medical education training session on management of severe pre-eclampsia and PPH, drills or simulations on preparation of parenteral drugs and preparation of patients for surgery that lasted half an hour, and displaying the Uganda clinical guidelines for management of severe pre-eclampsia in areas of care. The assumptions in this study were that there would be an improvement in adherence to standards of care after the intervention and also that the effect of confounders would be negligible.

The study population was the inpatients in St. Francis Hospital Nsambya who were diagnosed with PPH, severe pre-eclampsia or eclampsia. These inpatients’ inpatient-files were utilized as respondents. Data was collected by reviewing the inpatient files; a pre-tested and serialized check list with the standards of care was used to check whether the care documented in the files was according to the stated standards of care. Inclusion criteria were; In-patient diagnosed with PPH; In-patient diagnosed with severe pre-eclampsia; In-patient diagnosed with eclampsia. Exclusion criteria were; In-patient signed against medical care; In-patient referred out or asked for referral to another hospital; In-patient received hydralazine or magnesium sulphate at referring health unit; Inpatient’s inpatient-file missing from the records office. Data was collected from the records office by a medical doctor after the Institutional Research Committee had granted permission; it was then double entered into a computer and analysed using IBM SPSS Statistics version 20.

During the initial audit, all maternity inpatient files of patients admitted with or developed a diagnosis of severe pre-eclampsia or eclampsia or PPH during the months of August 2014, September 2014 and October 2014 were retrieved. The files of patients who met the exclusion criteria were excluded to remain with 67 files for severe pre-eclampsia/eclampsia and 58 files for PPH, making a total sample of 125 participants in the initial audit. The intervention was carried out in November 2014 and therefore no files were sampled during this month that was the intervention period.

During the re-audit, all maternity inpatient files of patients admitted with or developed diagnoses of severe pre-eclampsia, eclampsia and PPH during the months of December 2014, January 2015 and February 2015 were retrieved; the files of patients who met the exclusion criteria were excluded to remain with 44 files for severe pre-eclampsia/eclampsia and 69 files for PPH, making a total sample of 113 participants in the re-audit. The total number of participants in the study was therefore 238 for both the initial audit and the re-audit.

During data analysis, in order to show differences in adherence to standards of care, Pearson Chi Square test was done to establish whether the differences in adherences of the initial audit and the re-audit were statistically significant. For this statistical test to be done, collective adherence rates for the whole 3 month period of the initial audit were compared with the collective adherence rates for the whole 3 month period of the re-audit. Any *p* value less than 0.05 was taken to be a sign of statistically significant difference in the adherences.

## Results

The results have been presented in tables and graphs in order to reveal the differences between the adherences to the standards of care in the initial audit and in the re-audit. The *p* values that reveal statistically significant differences have been presented in the Table 2. Line graphs were plotted to show monthly variations in the adherence before the intervention (initial audit) and after the intervention (re-audit). The line in the centre of the graph shows the time when the intervention was done. Improvement is revealed by the line graphs when the graph for the re-audit is at a higher level which is also called a higher intercept, or when the slope of the graph for the re-audit changes [8]. Graphs were plotted for each standard of care, but they clearly showed improvement in adherence to 6 out of the 14 standards of care.

**Table 2:** Comparison of adherence rates of initial audit and re-audit for standards of care for severe pre-eclampsia/eclampsia and PPH

Standard of care	Adherence rate in initial audit (percentage)	Adherence rate in re-audit (percentage)	*P* value
Severe pre-eclampsia			
1. Patients should be seen by a doctor within 1 h of arrival.	58/67 (86.7)	43/44 (97.7)	0.045
2. Anti-hypertensive therapy should be started within 20 min of diagnosis.	13/67 (19.4)	30/44 (68.2)	<0.001
3. Urine protein dipstick test should be done within 30 min	50/67 (74.6)	36/44 (81.8)	0.375
4. BP should be monitored every 30–60 min when the diastolic BP is ≥110 mm Hg.	10/43 (23.3)	27/34 (79.4)	<0.001
5. The FHR should be monitored every 30 min when the diastolic BP is ≥110 mm Hg.	1/37 (2.7)	9/24 (37.5)	0.002
6. Magnesium sulfate should be administered within 20 min. of diagnosis	13/67 (19.4)	29/44 (65.9)	<0.001
7. A FBC, RFT and LFT should be done within 24 h	55/67 (82.1)	41/44 (93.2)	0.095
8. Steroid therapy should be given in all pregnancies where the pregnancy is estimated to be 28–34 weeks gestation.	9/12 (75.0)	7/8 (87.5)	0.769
9. Deep tendon reflexes test and respiratory rate monitoring should be done for 24 h	53/67 (79.1)	43/44 (97.7)	0.005
10. CS should be done in 1h from when decision is made	4/28 (14.3)	10/27 (37.0)	0.018
PPH			
1. AMTSL should have been done.	54/58 (93.1)	66/66 (100.0%)	0.026
2. IV oxytocin should be given as soon as possible	45/58 (77.6)	57/69 (82.6)	0.478
3. IV isotonic crystalloid fluids should be given as initial fluid resuscitation	50/58 (86.2)	67/69 (97.1)	0.023
4. In case bleeding does not respond to oxytocin, IV ergometrine or misoprostol should be given	48/54 (88.9)	38/41 (92.7)	<0.001

Table 2 shows that improvement was statistically significant for adherence to 7 of the 10 standards of care for severe pre-eclampsia. The 7 standards are: Patients should be seen by a doctor within 1 h (hour) of arrival; Anti-hypertensive therapy should be started within 20 min of diagnosis; BP (Blood Pressure) should be monitored every 30–60 min when the diastolic BP is ≥110 mm Hg; FHR (Fetal Heart Rate) should be monitored every 30 min when the diastolic BP is ≥110 mm Hg; Magnesium sulfate should be administered within 20 min. of diagnosis; Deep tendon reflexes test and respiratory rate monitoring should be done for 24 h; CS (Caesarean Section) should be done in 1h from when decision is made. As shown, the improvement was statistically significant for 4 of these 7 standards where the *p* value was less than 0.001 or was 0.002.

The table also shows that the improvement was statistically significant for 3 of the 4 standards of care for PPH. The 3 standards are: Active management of third stage of labour should have been done; IV (Intravenous) isotonic crystalloid fluids should be given as initial fluid resuscitation; In case bleeding does not respond to oxytocin, IV ergometrine or misoprostol should be given. The improvement was statistically significant for the standard of giving ergometrine or misoprostol because the *p* value was less than 0.001.

**Fig. 1:**
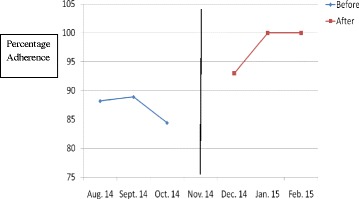
Adherence to severe pre-eclampsia patients being seen by a doctor within 1 h from arrival

**Fig. 2:**
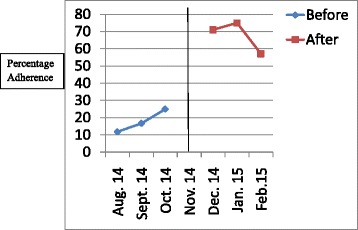
Adherence to starting antihypertensive therapy within 20 min. of diagnosis

**Fig. 3:**
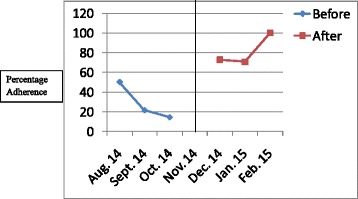
Adherence to BP monitoring every 30-60 min. when diastolic BP is ≥ 110 mmHg

**Fig. 4:**
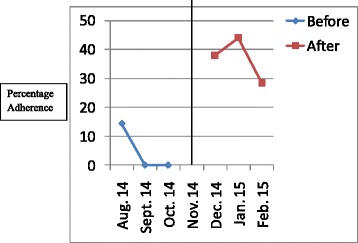
Adherence to FHR monitoring every 30 min. when diastolic BP is ≥ 110 mmHg

**Fig. 5:**
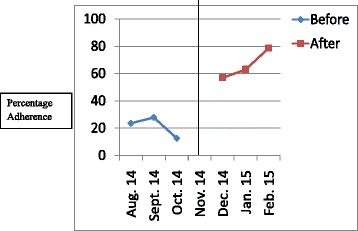
Adherence to magnesium sulphate administration within 20 min. of diagnosis

**Fig. 6:**
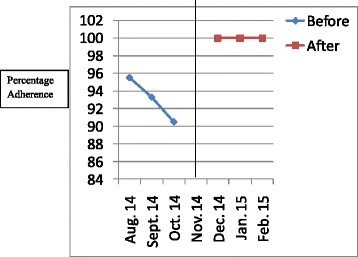
Adherence to doing active management of third stage of labour

Figures 1, 2, 3, 4, 5, 6 show the graphs for the 6 standards of care; 5 were for severe pre-eclampsia/eclampsia while 1 was for PPH. The figures show that the graphs for adherence after the intervention are at higher levels or intercepts and therefore, there was improvement in adherence to these 6 standards.

## Discussion

The initial audit revealed low adherence rates towards several guideline standards of care while the re-audit revealed improvements in adherence towards most of the standards of care. Several adherence rates were found below 50% in the initial audit and they improved to above 50% in the re-audit. The adherence rate for monitoring FHR every 30 min. when the diastolic BP is at least 110mmHg was still less than 50% in the re-audit, but the improvement in adherence was still found to be statistically significant. This is a sign that the interventions of CME, drills and displaying guidelines, as recommended during the audit cycle, were associated with improvement in quality of care.

Similarly, previous studies that involved carrying out CBA have shown that the implementation of recommendations made during the audit cycle were associated with improvements in quality of care. In Mulago Hospital, after an initial audit, one of the recommendations made and implemented was the displaying of guidelines and improvement in quality of care was realized [6]. In Muhimbili National Hospital, Dar es Salaam, one of the recommendations made after an initial audit was displaying guidelines and again quality of care improved [7]. The prevalence of severe PPH was found to reduce when feed-back to health workers concerning quality of care was the only intervention done [9]. In Malawi, implementation of recommendations that included both training health workers and displaying guidelines also resulted into improved quality of care [10].

There are a variety of interventions that can be done to effect behavior change among health workers with the goal of improving quality of care and these include training to give knowledge and skills, team building sessions and incentives [11]; however team building has been found to be a key component of behavior change [12, 13]. A combination of all is important for effective quality improvement. In this study, team building interventions like staffing and staff incentives were not done; these are interventions that can be done by hospital management to further effect change and it would be best to have included them. Availability of drugs and medical supplies was also assumed to be good yet in case it is not satisfactory; management would need to improve it in order to ensure good quality of care. The intervention in this study comprised training health workers and this is important for improving quality of care for patients [14]. CME is a tool for equipping health workers with knowledge [15] while displaying guidelines helps them to have something to refer to while providing care [16]. The use of drills during training helps the health workers to be prepared for the management of obstetric emergencies at all times [17]. Giving feedback was also done because it is also effective, but it is not very effective when performed in isolation [9].

There were 4 standards of care for which there was no statistically significant improvements in adherence after the intervention; 3 were standards of care for severe pre-eclampsia and 1 was a standard of care for PPH. These were; doing a urine protein dipstick test within 30 min, doing FBC (Full Blood Count), LFT (Liver Function Tests) and RFT (Renal Function Tests) within 24h, giving steroid therapy when the pregnancy is estimated to be 28-34 weeks of gestation, giving IV oxytocin as soon as possible when PPH is diagnosed. There are many factors that affect use of a guidelines, for example working conditions, self belief that improvement is possible and extent of staff shared vision [18]. It is however very likely that improvement was not significant for these standards because they had high adherence rates before the intervention leaving little room for improvement; some system factors can explain the high adherence rates, for example, all pregnant inpatients at this hospital have a urine protein dipstick test done immediately on admission irrespective of their condition. Additional reasons could be the short training period and use of few interventions – other additional interventions could have been team building sessions and increasing availability of drugs and supplies [6]; there could also be other reasons that the health workers know such that the best way to find out is by qualitatively interviewing them [19].

Among the quasi-experimental study designs, an interrupted time series is the strongest at minimizing the effect of confounders; a quasi-experimental design was the best for studying the effect of the intervention because of ethical problems associated with withholding such an intervention in a health unit [20]. The limitations of this study included the retrospective nature of data collection, the Hawthorne effect, the small numbers of patients, and the inability of the time series design to control for effects of history. However, the study was able to provide reliable information on how to improve quality of care while managing obstetric emergencies.

The retrospective nature of data collection could have affected the results because some of the inpatient-files could have been absent in the records office, and some of the data could have not been documented by the health workers. To minimize this limitation, the records staff were requested to organize the records at least one month prior to data collection; the denominators used when calculating the percentages of the indicators were adjusted to suit the number of files found to have the data needed.

Among the biases that could be present, one is as a result of the Hawthorne effect where the staff being aware of the ongoing study could have recorded better patient management after the intervention to give an impression of having improved yet in reality there could have been no improvement [21]; this was minimized by collecting data from the records office rather than on the wards where care is being given. Another bias that could be present is the one arising from the fact that the same medical doctor collected both the data before and after the intervention. The interrupted time series design itself cannot control for biases arising from occurrences in the hospital that can cause improvement in quality of care without being part of the intervention, and this could confound the results; this is also called inability of the design to control for effects of history.

The findings of this study show that when feedback on quality of care was given to the health workers and the recommendations of CME, drill and displaying of guidelines were implemented, the adherence to standards of care improved and hence the quality of care for PPH, severe pre-eclampsia and eclampsia. This improvement occurred without interventions like team building and improving availability of drugs and supplies upon which we the investigators had no control.

## Conclusions

There were improvements in the quality of care for patients with PPH, severe pre-eclampsia and eclampsia after giving feedback and implementing recommendations made following an audit of care. CBA is an effective method of improving quality of care in a health unit; it is very effective if recommendations made are implemented.

## Abbreviations

BP: Blood Pressure
CBA: Criteria Based Audit
CME: Continuing Medical Education
CS: Caesarean Section
FBC: Full Blood Count
FHR: Fetal Heart Rate
H: Hour
IV: Intravenous
LFT: Liver Function Tests
PPH: Postpartum Haemorrhage
RFT: Renal Function Tests

## References

[1] Say L, Chou D, Gemmill A, Tuncalp O, Moller A, Daniels J (2014). Global causes of maternal death: a WHO systematic analysis. Lancet Global Health.

[2] World Health Organisation (2012). WHO recommendations for the prevention and treatment of postpartum haemorrhage.

[3] World Health Organisation (2011). WHO recommendations for prevention and treatment of pre-eclampsia and eclampsia.

[4] World Health Organisation (2004). Beyond the Numbers: Reviewing maternal deaths and complications to make pregnancy safer.

[5] Pirkle C, Dumont A, Zunzunegui M (2011). Criterion-based clinical audit to assess quality of obstetrical care in low- and middle-income countries: a systematic review. International Journal of Quality in Health Care.

[6] Weeks A, Alia G, Ononge S, Otolorin E, Mirembe F (2005). A criteria-based audit of the management of severe pre-eclampsia in Kampala, Uganda. Int J Gynaecol Obstet.

[7] Kidanto H, Wangwe P, Kilewo C, Nystrom L, Lindmark G (2012). Improved quality of management of eclampsia patients through criteria based audit at Muhimbili National Hospital, Dar es Salaam, Tanzania: Bridging the quality gap. BMC Pregnancy and Childbirth.

[8] Penfold R, Zhang F (2013). Use if interrupted time series analysis in evaluating health care quality improvements. Academic Pediatric.

[9] Dupont C, Deneux-Tharaux C, Touzet S, Colin C, Bouvier-Colle M, Lansac J (2011). Clinical audit: a useful tool for reducing severe postpartum haemorrhage?. Int J Qual Health Care.

[10] Kongnyuy E, van den Broek N (2008). Criteria for clinical audit of women friendly care and providers’ perception in Malawi. BMC Pregnancy and Childbirth.

[11] Straus S, Tetroe J, Graham I (2009). Defining knowledge translation. CMAJ.

[12] Siassakos D, Draycott T, Crofts J, Hunt L, Winter C, Fox R (2010). More to teamwork than knowledge, skills and attitude. BJOG.

[13] Siassakos D, Bristowe K, Draycott T, Angouri J, Hambly H, Winter C (2011). Clinical efficiency in a simulated emergency and relationship to team behaviours: a multisite cross-sectional study. BJOG.

[14] Siassakos D, Crofts J, Winter C, Weiner C, Draycott T (2009). The active components of effective training in obstetrics emergencies. BJOG.

[15] Davis D, Davis N (2010). Selecting educational interventions for knowledge translation. CMAJ.

[16] Wensing M, Bosch M, Grol R (2010). Developing and selecting interventions for translating knowledge to action. CMAJ.

[17] Siassakos D, Hasafa Z, Sibanda T, Fox R, Donald F, Winter C (2009). Retrospective cohort study of diagnosis-delivery interval with umbilical cord prolapse: the effect of team training. BJOG.

[18] Durlak J, DuPre E (2008). Implementation Matters: A Review of research on the influence of implementation on program outcomes and the factors affecting implementation. Am J Community Psychol.

[19] Straus S, Tetroe J, Graham I, Zwarenstein M, Bhattacharyya O, Shepperd S. Monitoring use of knowledge and evaluating outcomes. CMAJ. 2010;10:1-5. 10.1503/cmaj.081335 PMC2817344 20083566

[20] Harris A, McGregor J, Perencevich E, Furuno J, Zhu J, Peterson D, Finkelstein J (2006). The use and interpretation of quasi-experimental studies in medical informatics. J Am Med Inform Assoc.

[21] Gale E (2004). The Hawthorne studies—a fable for our times?. Q J Med.

